# United Reference Method for three-dimensional treatment evaluation

**DOI:** 10.1186/s40510-018-0242-0

**Published:** 2018-12-03

**Authors:** Shereef Shahen, Manuel O. Lagravère, Gennaro Carrino, Fady Fahim, Reham Abdelsalam, Carlos Flores-Mir, Letizia Perillo

**Affiliations:** 1 Department of Orthodontics, University of Campania Luigi Vanvitelli, Naples, Italy; 2 Orthodontic Graduate Program, University of Alberta, Edmonton, AB Canada; 3 Department of Orthodontics, Cairo University, Cairo, Egypt; 4 Department of Orthodontics, Al Azhar University, Cairo, Egypt; 5 Head of the Division of Orthodontics and Orthodontic Program Director, Department of Dentistry, University of Alberta, Edmonton, Canada; 6 Head of Orthodontic Division and Chair of the Postgraduate Orthodontic Program, Department of Orthodontics, University of Campania Luigi Vanvitelli, Via L. De Crecchio 6, 80138 Naples, Italy

**Keywords:** Three-dimensional treatment evaluation, Descriptive three-dimensional superimposition, Three-dimensional reference system, Semi-automated landmarks

## Abstract

**Background:**

Reproducible and descriptive Three-dimensional treatment evaluation can enhance future treatment based on realistic results. So, the purpose of this study was to describe a new method for three-dimensional treatment evaluation showing how to use fully-automated craniofacial superimposition of CBCT records combined with reference system to obtain descriptive and comparable results. This new method was named United Reference Method (URM).

**Methods:**

URM is a combination of automated 3D superimposition on anterior cranial base surface anatomy and measurements based on reference system. It was developed to show how to use fully-automated superimposition to obtain descriptive numerical comparable values. The method is based on: one main reference system for both superimposed CBCT records, semi-automation to increase accuracy, all measurements are projections and auxiliary references to aid in landmarks identification and measurements.

The method steps can be described following a four-step approach: (1) Superimposition performed through a fully automated, voxel-wise, rigid registration considering only cranial base as a stable structure; (2) Identification of reference landmarks once on the superimposed records for corrected Frankfort Horizontal plane (C-FH) construction and a new semi-automated constructed Sella point to correct Orbital asymmetry; (3) Head orientation of superimposed CBCT images based on the C-FH; (4) Identification of landmarks affected by treatment with the aid of auxiliary reference planes. Evaluation of linear or angular changes derived by projection of same pre- and post-treatment landmarks on the C-FH. Pre- and post-expansion CBCT scans of 20 unilateral cleft lip and palate patients were used to calculate intra and inter-rater reliability. (X, Y and Z) coordinates, mean, standard deviation (SD) and Intra-class Correlation Coefficient (ICC) were calculated.

**Results:**

The proposed coordinates for C-FH construction showed ICC ≥ 0.998 and SD ranging from 0.064 to 0.242 mm. On the other hand, excluded coordinates due to expected natural craniofacial asymmetry had the lowest reliability ICC ≥0.742 and SD dramatically increased up to 1.112 mm.

**Conclusion:**

URM showed adequate reliability so it can be used to produce three-dimensional descriptive data of craniofacial structural changes.

## Background

Reproducible and descriptive treatment evaluation can provide realistic results. The obtained information can enhance future treatment. So, record as lateral cephalometric radiography was used for orthodontic diagnosis and treatment planning [1]. Although helpful, this imaging modality, it is a two-dimensional (2D) representation of a three-dimensional (3D) object and thus has several limitations, including errors in projection, distortion and structural superimposition, especially when used to evaluate skeletal and dental changes during treatment and/or growth. Despite these drawbacks, 2D records have been used over the last 80 years for superimposition and treatment change evaluation based on best fit of cranial base structures.

With the introduction of 3D imaging tools such as dental cone-beam computed tomography (CBCT), most limitations in 2D imaging have been theoretically overcome [2]. Many research groups have been developing 3D analysis based on CBCT superimposition to analyze treatment and/or growth changes [3–7]. Currently, two main methods developed by two different research groups are used to superimpose CBCT images. One group designed an optimization algorithm landmark-based superimposition approach [3]. This method identifies several landmarks to create a 3D coordinate reference system. Although effective, it presents several limitations such as errors made by the operator in locating the initial landmarks. The second group used a software voxel-based superimposition method with the best fit of the cranial base structures with high accuracy [4–6]. However, when using this approach, the descriptive amount and the spatial direction of changes are difficult to interpret. The main trend in the literature [2–6] was always to check 3D superimposition accuracy rather than to show how to use fully-automated superimposition to obtain descriptive numerical comparable values.

The aim of this study was to describe a new method for three-dimensional treatment evaluation showing how to use fully-automated craniofacial superimposition of CBCT records combined with reference system to obtain descriptive and comparable results. This new method was named United Reference Method (URM).

## Methods

Ethical approval was obtained from both of University of Campania “Luigi Vanvitelli” (Naples, Italy; approval number 1394/18) and Faculty of Oral & Dental Medicine, Cairo University (Cairo, Egypt; approval number 16/12/21).

URM is a combination of automated 3D superimposition on anterior cranial base surface anatomy and measurements based on reference system. The four-step URM approach as follows:

- Step 1 – Superimposition

Pre- and post-treatment CBCT scans were obtained using the same machine [8], parameters, x-ray intensity, imaging time and voxel size. In this study, SCANORA®3D (Soredex-Nahkelantie160, Tuusula, Finland) was used at 15 MA, 85 KV and 20 s exposure time. Digital Imaging and Communication of Medicine (DICOM) images were generated and exported with the same thickness (0.35), bit-depth (16) and dimensions (414X414 mm). The DICOM files were imported into Viewbox 4.0.1.7 software (dHAL Software, Kifissia, Greece, Athens) which has Computer-aided design (CAD) tools. 2D slices were assembled into volumes, subsequently converted into triangular mesh surface models created from the voxel data [4, 5, 9]. A different color was given to each record.

The pre- and post-treatment CBCT scans were superimposed using structures not displaced or changed during craniofacial growth or orthodontic treatment. This superimposition was performed using a fully automated, voxel-wise, rigid registration in the cranial base [4, 5, 10–13]. Cranial base registration was obtained by maximizing mutual voxel information from pre- and post-treatment CBCT images to avoid inevitable observer-dependent error when using techniques based on overlap of anatomic landmarks [4].

As this superimposition depends on masking or eliminating structures that may be affected by treatment or growth, so that the software searched only in cranial base structures. Leaving other structures, affected by treatment and/or growth changes, would otherwise increase the working time required by the software to search for all available possibilities to find the best match [4, 5]. The structures used as reference for the cranial base included anterior part of hypophyseal fossa, wings of spenoid, crista galli. The software provides tools to select 3D zone useful for superimposition in order to exclude parts subjected to change. Reproducibility of this masking method was already reported in literature [4, 5, 14].

After superimposition was completed, maxillary and mandibular structures were unmasked. The two CBCT scans were then recorded as linked files and treated as one*.* Some parts of the two CBCT images appeared fused and indistinguishable, suggesting the high quality of the automated superimposition whereas the non-fused parts indicated changes. Mesh images are necessary for superimposition, later any 3D display (multiplanar reconstruction, direct volume rendering, isosurface and mesh) can be used.

- Step 2 – Reference landmark identification

**Fig. 1:**
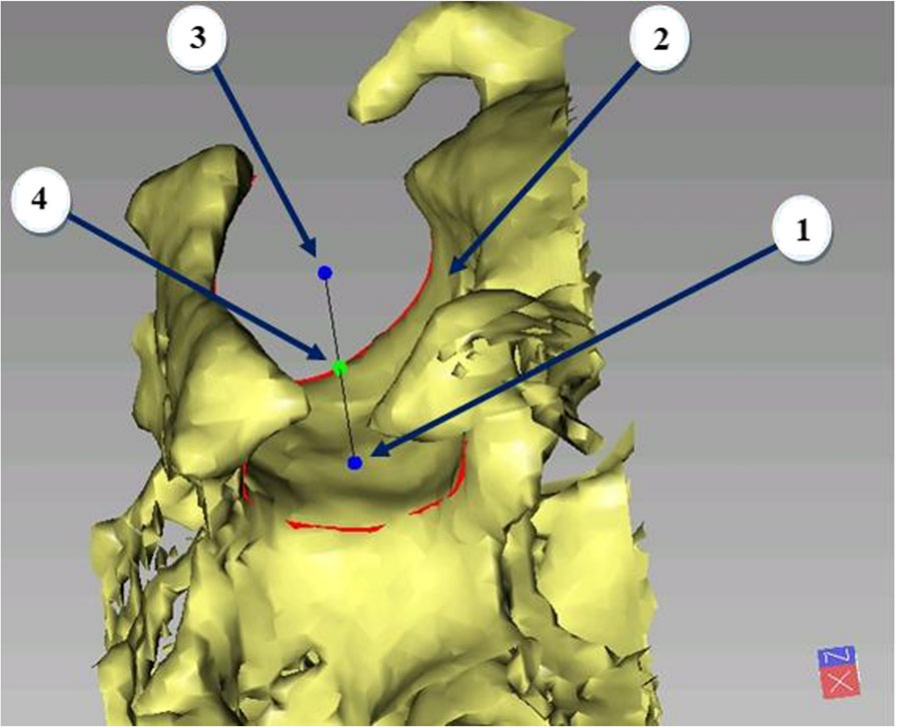
Constructing R-and L-Sella: 1, R-Sella; 2, Hypophyseal fossa; 3, L-Sella; 4, C-Sella. Red line: Sella border

Reference landmarks in Table 1 [15, 16] were identified once on the superimposed pre-treatment CBCT record to construct Corrected Frankfort Horizontal plane (C-FH), the new C-Sella (Fig. 1) and Porion axis were used to correct Orbital asymmetry producing new C-Orbitale points.

**Table 1:** Landmark definition for use in reference system

Landmark	Definition
Right Sella and Left Sella (R- and L Sella)	Points in the center of the right and left 3D borders of the Hypophyseal fossa. Points are digitized semi-automatically through manual tracing of the right and left borders of the Hypophyseal fossa and then automatically digitized by the software. (Fig. 1)
Semi-automated Constructed Sella (C-Sella)	A constructed semi-automated point that lies midway between right and left Sella. (Fig. 1)
Porion (Po)	The most superior midpoint of each external acoustic meatus.
Porion axis	A line passing between the two Porion points. (Fig. 2)
Orbitale (Or)	The most inferior point of each infra-orbital rim.
Mid-Orbitale	A point midway between the two Orbitale points. (Fig. 2)
Right and Left Constructed Orbitale (C-Orbitale)	Points at the level of mid-Orbitale point superior-inferiorly (Z coordinate) and lies at equal distances medio-lateraly from the C-Sella point (X coordinate), in a parallel direction to the Porion axis (Y coordinates). (Fig. 2)

**Fig. 2:**
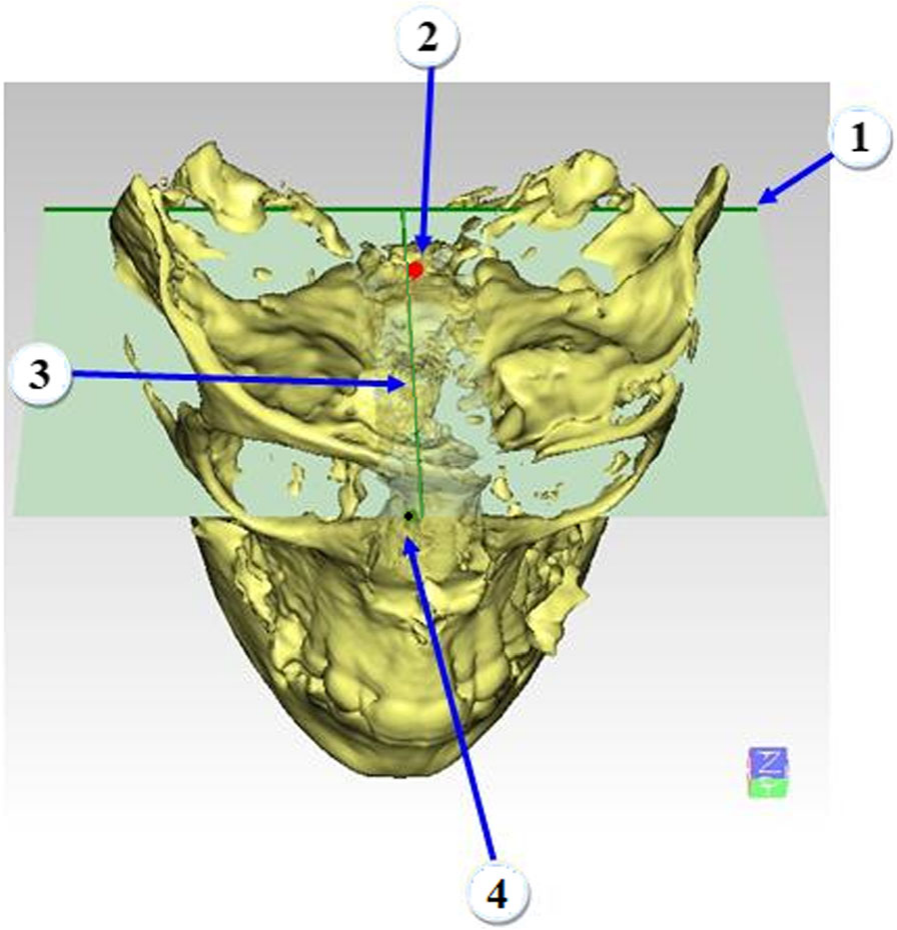
C-FH:1, Porion axis; 2, C-Sella; 3, Midline; 4, Mid Orbital point

The C-FH was constructed from four points: right and left Porion points, and right and left C-Orbitale points and midline was defined by C-Sella and Porion axis to correct Frankfort Horizontal (FH) midline (Figs. 1 and 2). C-FH was used for head orientation and to produce projection measurements describing numerically direction of changes whereas it was not used for superimposition, which is fully-automated.

- Step 3 – Head orientation

After locating reference landmarks, the superimposed CBCT scans were reoriented to make C-FH horizontal using the software. Orientation was required at this point to create new coordinate system with zero point at the center of C-FH in addition to standardize the 3D view and to reduce errors in identifying landmarks necessary for assessing changes [15, 17].

- Step 4 – Identification of landmarks for evaluating changes

**Fig. 3:**
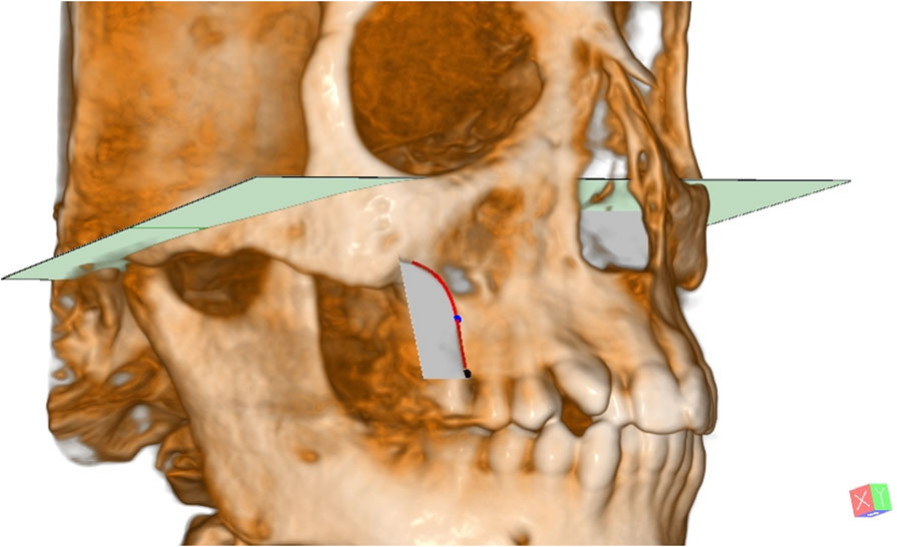
Maxillary basal bone. Red curve shows cut at Mid-Max. Perpendicular plane. Blue point is R-Max. Black point is R-ALV

Landmarks to determine 3D skeletal and dental changes in Table 2 during treatment or growth were identified on the reoriented CBCT images with the aid of auxiliary reference planes the mid-maxillary perpendicular plane was constructed cutting the maxilla perpendicularly to the C-FH midway between ANS and PNS points. R- and L-Max can be digitized semi-automatically by manual tracing of the right and left maxillary cortical bone on a cut at the level of Mid Max Perpendicular plane level. The most concave point was automatically digitized at the nearest point to the midline in relation to C-FH (Fig. 3). These landmarks need to be located twice and only after head orientation (step 3), unlike reference landmarks previously identified once in step 2.

**Table 2:** Landmark definition for structures affected by treatment

Landmark	Definition
ANS	The most anterior midpoint of the anterior nasal spine of the maxilla.
PNS	The most posterior midpoint of the posterior nasal spine of the palatine bone.
R-Max and L-Max	The Most concave point on the maxillary basal bone on the Mid-Max. Perpendicular plane. (Fig. 3)
R- Alveo and L- Alveo	The most inferior point of the alveolar crest on the Mid-Max. Perpendicular plane. (Fig. 3)
Molar axis	The line perpendicular on the plane constructed from the three molar root apices.
Premolar axis	The line from the buccal cusp tip to the apex of buccal root of the upper first premolar.

**Fig. 4:**
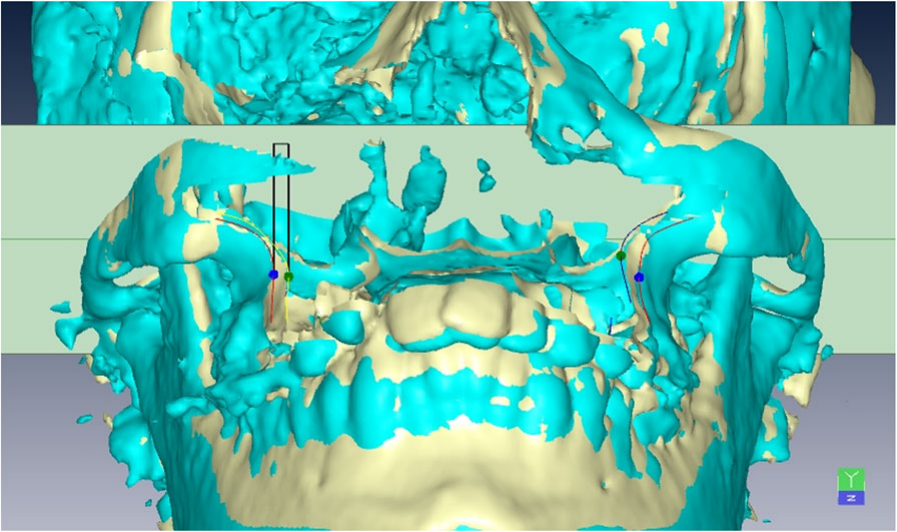
Distance traveled by R- Max mediolaterally. Green point: Pre-expansion R-Max & L-Max. blue point: Post expansion R-Max & L-Max

Linear and angular measurements obtained from the same points identified in pre- and post-treatment CBCT images, (e.g., pre-and post-treatment R- and L-Max), were projected in a direction parallel or perpendicular to C-FH (Fig. 4). To show how to evaluate linear and angular changes after expansion treatment of cleft patients using URM, pre- and post-expansion (after 6 months) CBCT images of 20 unilateral cleft lip and palate patients (mean age of 20 years ranging between 18 and 25) were collected from the archives of Department of Orthodontics, Cairo University, Cairo, Egypt. The ALARA principle (radiation dose ‘As Low As Reasonably Achievable’) was respected. Post-expansion CBCT records, required to evaluate the cleft space for the alveolar bone grafting, were also useful to measure the achieved expansion in the cleft patients. Measurement definitions were reported in Table 3. All previous steps were to explain to developers but actual steps carried out by the users are: first masks structures may be affected by treatment then click superimposition, second identifies reference points R-L Sella, R-L Porion and R-L Orbitale, third click to orient superimposed pre- and post-treatment CBCTs. Fourth identify points affected by treatment then save results.

**Table 3:** Definition of measurements for structures affected by treatment

Parameters		Definition
Linear Measurements (mm)	R-Max and L-Max	Distance traveled by Max mediolaterally. (Fig. 4)
	R-Cres and L-Cres	Distance traveled by Alveo mediolaterally.
Angular Measurements (°)	R-ALV and L-ALV	Angular change between pre and post lines from Max to Alveo projected on Mid Max Perpendicular plane, perpendicular to C-FH.
	R-Pre and L-Pre	Angular change between pre and post premolar axes projected on Mid Max Perpendicular plane, perpendicular to C-FH.
	R-6 and L-6	Angular change between the pre and post molar axes projected on Mid Max Perpendicular plane, perpendicular to C-FH.

### Reliability of landmark location and CBCT orientation

The obtained CBCT records were used also to calculate intra- and inter-rater reliability for C-FH. R-L Sella, C-Sella, R-L Po and R-L Orbitale were identified three times at weekly intervals by three observers (SS, FF, RA). Three-dimensional coordinates (X, Y and Z based on the original volume coordinate system before orientation) were exported to an Excel spreadsheet before carrying out a statistical analysis. Mean, standard deviation (SD) and Intraclass Correlation Coefficient (ICC) were calculated to evaluate the reproducibility of the newly C-FH but not the automated-superimposition which was already proven to be accurate [4–6].

## Results

Three coordinates (X, Y and Z coordinates) were needed to determine the spatial position of each point. Constructed Orbitale point was identified based on Z coordinate of mid-Orbitale point, X coordinate of C-Sella and Y direction of Porion. The following coordinates: X coordinate of C-Sella, Z coordinates of Porion and Orbitale, Y coordinate of Porion, used to construct C-FH and correct natural craniofacial asymmetry had good reliability: ICC ≥ 0.998 (Table 4, Figs. 1 and 2). On the other hand, the excluded coordinates due to expected natural craniofacial asymmetry (X coordinates of Porion and Orbitale and Y coordinate of Orbitale) had the lowest reliability: ICC ≥ 0.742 and ICC ≥ 0.967, respectively.

**Table 4:** Intraclass Correlation Coefficient (ICC) and Standard deviation (SD) of Intra- and Inter- rater reliability error for landmarks digitization of X, Y and Z coordinates for Reference system

Landmark		Intra-rater						Inter-rater					
		X		Y		Z		X		Y		Z	
		ICC	SD	ICC	SD	ICC	SD	ICC	SD	ICC	SD	ICC	SD
C-SELLA	Single Measures	1	0.070	1	0.014	1	0.032	0.998	0.064	0.999	0.238	1	0.108
	Average Measures	1		1		1		0.999		1		1	
R-SELLA	Single Measures	1	0.097	1	0.0359	1	0.026	0.992	0.116	0.999	0.220	1	0.126
	Average Measures	1		1		1		0.997		1.000		1	
L-SELLA	Single Measures	1	0.029	1	0.023	1	0.052	0.996	0.093	0.999	0.254	1	0.116
	Average Measures	1		1		1		0.999		1		1	
R-PORION	Single Measures	0.994	0.497	1	0.102	1	0.083	0.945	0.724	0.998	0.195	0,999	0.242
	Average Measures	0.998		1		1		0.981		0.999		1	
L-PORION	Single Measures	0.990	0.788	1	0.180	1	0.172	0.995	0.618	1	0.258	0,998	0.172
	Average Measures	0.997		1		1		0.998		1		0,999	
R-ORBITALE	Single Measures	0.999	0.229	0.999	0.305	1	0.055	0.742	0.980	0.982	0.462	0,999	0.170
	Average Measures	1		1		1		0.896		0.994		1	
L-ORBITALE	Single Measures	0.994	0.200	0.996	0.676	1	0.108	0.915	1.112	0.967	0.532	0,999	0.159
	Average Measures	0.998		0.998		1		0.97		0.989		1	

The proposed coordinates showed standard deviations ranging from 0.064 mm in X coordinate of C-Sella to 0.242 mm in Z coordinate of Porion. In addition, Z was the only proposed coordinate of Orbitale with a standard deviation ranging from 0.055 mm to 0.170 mm while the standard deviation of excluded coordinates dramatically increased up to 1.112 mm as in X coordinate of Orbitale (Table 4, Figs. 1 and 2). The linear and angular changes after expansion treatment of the unilateral cleft lip and palate patients are shown in Appendix 1.

## Discussion

During the last decades, superimposition based on either landmarks or structures has been used to visualize craniofacial changes. After reaching adequate accuracy level following automation of 3D structural superimposition, these changes can now be quantified and described three-dimensionally. However, this process is challenging and prone to many errors [4, 5, 14]. This study proposes a more effective way to use automated superimposition to describe 3D changes. Some Authors used teeth as references [18–20], but teeth can move and are thus unreliable landmarks. To overcome this issue, stable references that do not change during growth and/or orthodontic treatment were selected [3].

Skeletal landmarks should be carefully selected to avoid unreliable reference landmarks located in anatomical structures subject to growth or treatment effects [3]. It has been suggested that cranial base landmarks located in anatomically stable structures can be identified from CBCT imaging with very good reliability, although it can be argued that more than 85% of cranial base growth is completed by age of five [3].

Another technical error is the use of direct measurements, such as the distance between two landmarks, which are unable to give information about the direction of changes [18, 19]. Such data can be obtained using the URM by projecting the measurements on a C-FH reference plane [17].

Currently, two main methods are routinely utilized to evaluate 3D treatment changes. The first method [5, 14] uses automatic voxel-based superimposition and changes are visualized by different color depictions indicating different changes. Although this method was proven to be reproducible [4–6, 21], it still presents some limitations as it can only produce direct measurements and thus no descriptive results [17]. Moreover, superimposition can only be used to compare pre- and post-treatment records of the same patient. It does not allow comparison of data derived from different patients due to the lack of a common reference system [7]. The second method [20] uses a reference system identified twice, once for pre- and once for post-CBCT images to take measurements on pre- and post-CBCT scans without superimposition with the advantage of showing direction of measurements. However, this method can likewise be prone to error because the required multiple and repeated landmark identification processes can increase the overall combined possibility of error [21]. Compared to 2D analysis, possible landmark location error increases in 3D analysis due to the presence of a third dimension-coordinate [22].

This study proposes how to use of 3D superimposition combining the advantages of the first and second methods. It was named United Reference Method (URM) because, after automatic superimposition, only one reference system is identified once and all the measurements are projected on C-FH after. This does not assume that reference will not change, creating unified coordinate system is the target to compare results between pre- and post-expansion records.

The advantage of digitizing reference landmarks only once is that it eliminates error related to reference. In other words, if an error cannot be avoided, the same error will be made on the superimposed CBCT records. This does not mean that the method is error free, but only that the error related to reference becomes constant and has no effect on evaluated changes. Nevertheless, even using URM, error related to areas affected by growth and/or treatment remains unavoidable since related points are identified twice.

In addition, semi-automation in landmark identification has further advantages. It can help to accurately identify challenging points. For example, the Hypophyseal fossa is not a uniform 3D cavity and so it is difficult to identify a reproducible point in the center without the help of software. Semi-automation can create new 3D points, which were not described before. For instance, the URM allowed for the identification of reproducible skeletal points, such as R- and L- Max-, on the lateral smooth surface of the maxilla (Fig. 3). Semi-automation can also enhance reference reliability utilizing C-Sella, which was shown to be reproducible. C-Sella reduces the impact of Orbitale asymmetry and corrects FH midline with also the help of Porion axis, improving FH construction. The new plane is named C-FH. In contrast, the use of the original Orbitale points could result in poor FH midline and twisted FH (Fig. 2). Even though Porion points may have a degree of natural asymmetry [23] this problem could be more obvious in the Orbitale points. It has to be noted that the URM midline was derived from C-Sella independent of X coordinates of Porion and Orbitale points. The generated points are based on low error coordinates only [24].

Another feature of the URM is its different use of slices. Several authors evaluate CBCT using slice direction derived from head orientation during or after imaging without a reliable standardization [17, 18]. In addition, the position of these slices is also related to unstable landmarks [17–19] In some 3D studies [1, 16, 18, 19, 25, 26], head orientation is based on operator evaluation of various structures to obtain a more horizontal slicing direction, whereas reference points are identified later. Thus, head orientation can be subject to a degree of variability and is not linked to subsequently identified references. Conversely, slice direction in the URM is derived from a standard head orientation obtained from C-FH, and slices linked to the C-FH may enhance reproducibility of point identification. Furthermore, measurements from 2D slices [18, 19] are combined with 3D display and standardized using a reference plane unaffected by treatment, as previously reported [3].

Some previous studies stated that metallic landmarks identification and cortical bone thickness measurements are not affected by imaging head position [8, 27, 28], whereas the amount of directional change is strongly influenced by head orientation [29].

This difference between the URM and other methods is depicted when evaluating the treatment of expansion cases, where direct measurements on 2D slices from spiral CT [26] were used, and by other papers applying 3D display without any reference [30] or with unstable reference [31, 32]. Semi-automation in URM allowed identification of reproducible skeletal points, such as R- and L-Max on the wide lateral surfaces of maxilla where are no sharp 3D skeletal landmarks can be identified. Thus, many researchers were obliged to use dental points to evaluate 3D skeletal expansion [18–20] even though dental points are unreliable landmarks. Conversely, the URM measured mediolateral change by projecting R-and L-Max identified on superimposed pre- and post-CBCT scans on C-FH (Fig. 4) derived from structures unaffected by treatment. In addition, with the URM, only one reading for each measurement was sufficient to produce a descriptive standardized result describing exactly how much basal bone of maxilla moved medio-laterally and in which direction projecting changes on C-FH. Thus, the operator can compare pre-and post-treatment results, simplifying data interpretation.

### Limitations

The URM has some limitations although not strictly related to the method but to all CBCT superimposition methods. Generating high quality CBCT images to make reliable superimpositions is still challenging. The same machine with the same imaging parameters for each patient may be more useful. Few imaging manipulation programs offers advanced geometric and CAD features, limiting the use of the URM. Necessity to make pre- and post-treatment CBCT records to perform superimposition.

Although common X-ray effective dose of CBCT was reduced to 50 μSv in comparison to 2000 μSv in conventional CT [33], exposure may be still considered an issue to keep exposure to radiation as low as is reasonably achievable (ALARA). This may be justified if CBCT was a substitution of all other radiological exams. “the sum of the effective doses for panoramic and lateral cephalometric and periapical images would be in the same range or even higher than that of CBCT, and still without 3D evaluation.” [34]. In addition, recent technology of Ultra Low Dose (ULD) can even reduce the effective dose to 18 μSv for 200 mm^3^ volume [35].

## Conclusion

- URM showed adequate reliability and could be used to produce three-dimensional descriptive data of craniofacial structural changes.
- Superimposition alone cannot provide descriptive measurements Thus, projected measurements on reference system is essential for meaningful readings.

## Appendix

**Table 5:** Linear and angular changes after expansion treatment of the unilateral cleft lip and palate patients

Patients	Linear measurements (mm)				Angular measurements (°)					
	R-Max	L-max	R-Cres	L-Cres	R-6	L-6	R-Pre	L-Pre	R-Alv	L-Alv
1	2.8	1.4	3.9	2.1	11.1	1.4	6.2	1.9	7.1	3.9
2	3.9	3.2	3.6	6	8.2	30.9	7.8	12.1	1.5	9.5
3	1.8	0.1	1.7	1.6	8.6	10.1	4.4	8.3	0.2	8.9
4	0.7	1.4	0.6	3.6	14.4	17.4	3.8	0.5	0.3	6.5
5	2.8	3.9	4.4	5.2	11.2	18.6	4.2	4	10.7	29.4
6	3.4	2.6	2.5	4.1	10	23	4.8	4.2	9.9	13.9
7	3	2.6	2	4.3	1.2	13.5	0.6	3.7	9.5	12.1
8	3	2.5	4.7	2.4	10.3	7.4	8.1	4	18.7	1.2
9	3.5	0.7	5.6	4.5	17.3	14.9	12.8	20.5	5.8	3.3
10	3.7	3.9	4.2	3.6	7.8	13	4.4	2	2.9	1.3
11	1.4	2.2	1.7	3.1	15.6	20.1	6.8	4.6	14.6	5.6
12	0.9	1	2.3	1.5	8.6	7.2	10	6.1	7	1.8
13	5.4	4	4.4	5	0.5	7.8	11.8	2.9	19.2	4.9
14	0.3	1.9	0.6	1.7	9.5	10.5	2.4	4.2	2.4	2.3
15	3.9	4	4.5	3.8	1.5	13	1.3	5.5	1.8	1.8
16	0.9	1.5	3	2.4	17.9	16.9	0.5	2.9	12	4.7
17	2.4	3.2	3.3	4.6	1.5	1.3	7.5	5.6	6.7	6.8
18	1.6	4	1.1	1.9	14.5	16.6	3.2	7	5.4	28.3
19	0.9	2.4	1.1	3.7	6.9	5.8	12.9	13.9	0	12.4
20	3.5	0.7	4.2	1.4	17.3	14.9	12.8	20.5	5.8	3.3

## Abbreviations

2D: Two-dimensional
3D: Three-dimensional
ALARA: Principle: Radiation dose ‘As Low As Reasonably Achievable’
CAD: Computer-aided design
CBCT: Cone-beam computed tomography
C-FH: Corrected Frankfort Horizontal plane
DICOM: Digital Imaging and Communication of Medicine
FH: Frankfort Horizontal
ICC: Intra-class Correlation Coefficient
SD: Standard deviation
URM: United Reference Method
